# The G protein‐coupled receptor ligand apelin‐13 ameliorates skeletal muscle atrophy induced by chronic kidney disease

**DOI:** 10.1002/jcsm.13159

**Published:** 2022-12-23

**Authors:** Yuki Enoki, Tomoya Nagai, Yuna Hamamura, Sumika Osa, Hideaki Nakamura, Kazuaki Taguchi, Hiroshi Watanabe, Toru Maruyama, Kazuaki Matsumoto

**Affiliations:** ^1^ Division of Pharmacodynamics Keio University Faculty of Pharmacy Tokyo Japan; ^2^ Faculty of Pharmaceutical Sciences Sojo University Kumamoto Japan; ^3^ Department of Biopharmaceutics Graduate School of Pharmaceutical Sciences, Kumamoto University Kumamoto Japan

**Keywords:** apelin, Apj, chronic kidney disease, elabela, skeletal muscle atrophy, uraemic toxin

## Abstract

**Background:**

Targeting of the apelin–apelin receptor (Apj) system may serve as a useful therapeutic intervention for the management of chronic kidney disease (CKD)‐induced skeletal muscle atrophy. We investigated the roles and efficacy of the apelin–Apj system in CKD‐induced skeletal muscle atrophy.

**Methods:**

The 5/6‐nephrectomized mice were used as CKD models. AST‐120, a charcoal adsorbent of uraemic toxins (8 w/w% in diet), or apelin (1 μmol/kg) was administered to CKD mice to investigate the mechanism and therapeutic potential of apelin on CKD‐induced skeletal muscle atrophy. The effect of indoxyl sulfate, a uraemic toxin, or apelin on skeletal muscle atrophy was evaluated using mouse myoblast cells (C2C12 cells) in vitro.

**Results:**

Skeletal muscle atrophy developed over time following nephrectomy at 12 weeks, as confirmed by a significant increase of atrogin‐1 and myostatin mRNA expression in the gastrocnemius (GA) muscle and a decrease of lower limb skeletal muscle weight (*P* < 0.05, 0.01 and 0.05, respectively). Apelin expression in GA muscle was significantly decreased (*P* < 0.05) and elabela, another Apj endogenous ligand, tended to show a non‐significant decrease at 12 weeks after nephrectomy. Administration of AST‐120 inhibited the decline of muscle weight and increase of atrogin‐1 and myostatin expression. Apelin and elabela expression was slightly improved by AST‐120 administration but Apj expression was not, suggesting the involvement of uraemic toxins in endogenous Apj ligand expression. The administration of apelin at 1.0 μmol/kg for 4 weeks to CKD mice suppressed the increase of atrogin‐1 and myostatin, increased apelin and Apj mRNA expression at 30 min after apelin administration and significantly ameliorated weight loss and a decrease of the cross‐sectional area of hindlimb skeletal muscle.

**Conclusions:**

This study demonstrated for the first time the association of the Apj endogenous ligand–uraemic toxin axis with skeletal muscle atrophy in CKD and the utility of therapeutic targeting of the apelin–Apj system.

## Introduction

Chronic kidney disease (CKD) is a global concern due to the lack of treatment options and associated complications including morbidity and mortality.^1^ Among the several complications associated with the progression of CKD, skeletal muscle atrophy is the most prevalent^2^ besides death caused by cardiovascular disease (CVD),^3^, ^4^ which is the leading cause of death as a result of CKD. It has been reported that low skeletal muscle mass and strength are closely associated with poor renal prognosis and an increase in mortality in CKD and dialysis patients.^5^, ^6^ This indicates the existence of vicious cycle mechanisms in the progression of kidney impairment and skeletal muscle atrophy under conditions of CKD and highlights the requirement of therapeutic strategies to target skeletal muscle atrophy and prevent CKD progression and death.

Among the several therapeutic interventions for CKD‐induced sarcopenia proposed over the last few decades, the apelin–apelin receptor (Apj) system has attracted some attention in recent years. Apj is known as the receptor for apelin and was identified and cloned as G protein‐coupled receptor (GPCR) in 1993,^7^ and after that, two endogenous ligands of Apj were identified—apelin, which was identified in 1998,^8^ and elabela, which was identified in 2013.^9^ The apelin–Apj system has been reported to be expressed in many organs, including skeletal muscle, kidney, lung, adipose tissue, heart and brain.^10^ Several studies have reported that apelin has physiological effects on skeletal muscle.^11^, ^12^ For example, apelin enhances glucose uptake via Akt phosphorylation in skeletal muscle.^11^ Furthermore, apelin increases mitochondrial biogenesis and protein synthesis via activation of AMPK, AKT and P70S6K.^12^ These signals activated by apelin are effective therapeutic targets in sarcopenia^13^ and, therefore, show the therapeutic potential of apelin on the loss of skeletal muscle mass and function. However, it has been reported that exercise increases the expression of apelin blood, but the findings regarding the efficacy of exercise‐induced apelin on sarcopenia are controversial and further studies are needed.^14^ This evidence indicates that intervention in the apelin–Apj could be a promising strategy for the treatment of skeletal muscle atrophy and the comprehensive management of CKD.

Thus, this study aimed to investigate the association between apelin and skeletal muscle atrophy in CKD and evaluate the potential of apelin as a therapeutic agent for skeletal muscle atrophy in CKD.

## Materials and methods

### Materials

AST‐120 (a charcoal adsorbent of uraemic toxin, Kremezin^🄬^) was purchased from Mitsubishi Tanabe Pharm Corp., Ltd. (Osaka, Japan). Indoxyl sulfate potassium (IS) was purchased from Santa Cruz Biotechnology (Dallas, TX, USA). Dulbecco's modified Eagle's medium (DMEM), heat‐inactivated horse serum and anti‐laminin antibody (#L9393) were purchased from Sigma‐Aldrich (St. Louis, MO, USA). Anti‐rabbit‐IgG‐Alexa594 (#A11012) was purchased from Invitrogen (Waltham, MA, USA). Superprep^🄬^ II cell lysis and the RT kit for qPCR were purchased from TOYOBO (Osaka, Japan). Foetal bovine serum (FBS) was purchased from Gibco (Grand Island, NY, USA). Human tumour necrosis factor‐α (TNF‐α) was purchased from Proteintech Group (Rosemont, IL, USA). 4‐hydroxynonenal was purchased from Cayman Chemical Co., Ltd. (Ann Arbor, MI, USA).

### Apelin synthesis

Apelin (QRPRLSHKGPMPF) was synthesized on a 2‐chlorotrityl chloride resin using the standard Fmoc solid‐phase methodology using a microwave synthesizer (Biotage® Initiator + Alstra, Tokyo, Japan). Details of the synthesis methods are described in the supporting information. The synthesized apelin was purified via high‐performance liquid chromatography (HPLC). The molecular weight of synthesized apelin was determined by matrix‐assisted laser desorption ionization time of flight mass spectrometry. Detail chromatograms for apelin determination were indicated in *Figure* *S1*.

### Animal experiments

**Figure 1:**
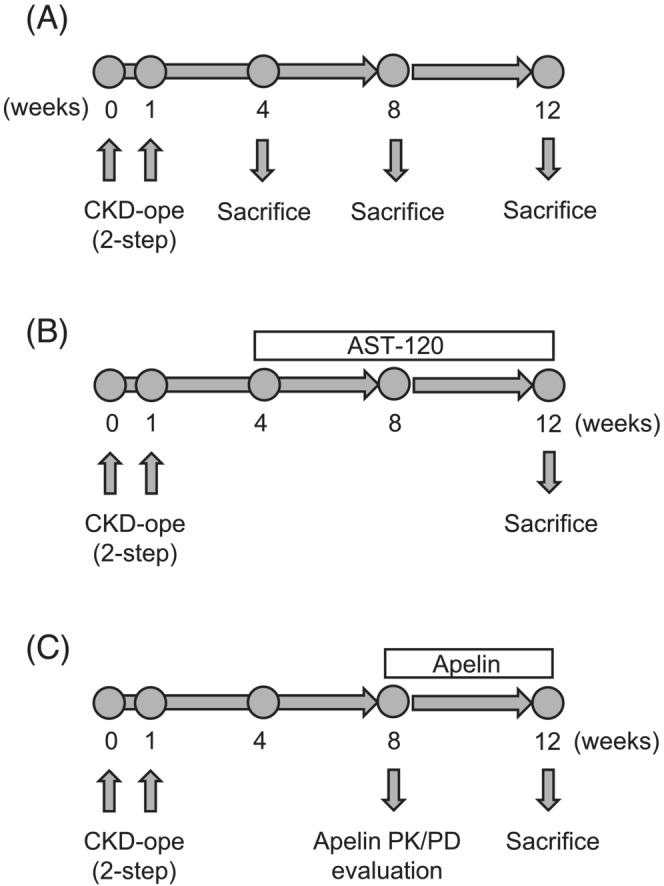
Experimental scheme. (A) Evaluation of skeletal muscle atrophy progression and alteration of myokine expression in chronic kidney disease (CKD) mice. 5/6‐Nephrectomy was performed by two‐step surgery, and mice were maintained with standard food and water. Mice were sacrificed and evaluation was performed at 4, 8 and 12 weeks after second‐step surgery. (B) Evaluation of relationship between uraemic toxins and myokine expression in skeletal muscles of CKD mice. At 4 weeks after 5/6‐nephrectomy, mice were randomized and AST‐120 containing powder diet (8 w/w%) was administered for 8 weeks and mice were then sacrificed and evaluation was performed. (C) Evaluation of effect of apelin on CKD‐induced skeletal muscle atrophy. At 8 weeks after 5/6‐nephrectomy, mice were administered apelin once a day (1 μmol/kg, intraperitoneally) for 4 weeks and then mice were sacrificed and evaluation was performed.

Male C57BL/6JJmsSlc mice (weight: 22–25 g) were purchased from Japan SLC Inc. (Shizuoka, Japan). Mice were maintained with free access to food and water and housed in a controlled room with 12 h day/night cycle and temperature (21–23°C). All animal experiments were performed in accordance with the protocols approved by the Institutional Animal Care and Use Committee of Keio University (approval number: 18015). 5/6‐Nephrectomy (Nx) two‐step surgery was performed as described in previous reports.^15^, ^16^ After 5/6‐Nx surgery, the mice were randomized and used in each experiment. In the AST‐120 treatment group, the mice were randomized by body weight and kidney function at 4 weeks after 5/6‐Nx, and AST‐120 containing powder diet (8 w/w%) was administered for 8 weeks. Serum creatinine (SCr) and blood urea nitrogen (BUN) levels were used as indices of kidney function. In the apelin‐administration experiment, the mice were randomized by body weight and kidney function at 8 weeks after the second surgery, and apelin was administered intraperitoneally once a day (1 μmol/kg) for 4 weeks. After drug administration, blood and skeletal muscle tissues were collected. The number of mice used in this study is as follows: Experimental Scheme 1 (*Figure* *1* *A*), sham *n* = 4, CKD *n* = 4; Experimental Scheme 2 (*Figure* *1* *B*), sham *n* = 6, CKD *n* = 8, CKD + AST‐120 *n* = 7; and Experimental Scheme 3 (*Figure* *1* *C*), CKD *n* = 6, CKD + apelin *n* = 5.

### Serum blood urea nitrogen, creatinine, apelin and indoxyl sulfate level

Serum concentration of BUN and creatinine was measured using FUJI DRI‐CHEM 7000 and a dry chem slide system (FUJIFILM, Kanagawa, Japan) as per the protocol described by the manufacturer. The serum apelin and elabela concentration was determined via enzyme‐linked immunoassays using an EIA Kit (Phoenix Pharmaceuticals, Inc., Burlingame, CA, USA) as per the protocol described by the manufacturer. The serum concentration of IS was measured via HPLC, as described previously, with a few modifications.^15^ The HPLC system consisted of a CBM‐20A communication bus module, SPD‐20A, a degasser and an LC‐20 AD pump (SHIMADZU Co., Kyoto, Japan). The conditions for HPLC were as follows: column, Unison UK‐C18 (3 μm, 250 mm × 4.6 mm).

### Cell culture

C2C12 myoblasts were purchased from the RIKEN BioResource Cell Bank (Ibaraki, Japan) and maintained in growth medium (GM), which consisted of DMEM, 10% FBS, 100 U/mL penicillin and 100 μg/mL streptomycin at 37°C and 5% CO_2_. C2C12 myoblasts were seeded (1 × 10^4^ cells per well) in a 96‐well plate. To induce myotube formation, the GM was switched to differentiation medium (2% heat‐inactivated horse serum containing DMEM) after cells were adherent and semi‐confluent (approximately 80–90%).

### Real‐time RT‐PCR analysis

RNA was isolated from the gastrocnemius (GA) muscle and cDNA was synthesized using the Superprep^🄬^ II as per the protocol described by the manufacturer. In brief, cells were lysed with lysis reagent supplemented with RNase inhibitor and genome DNA remover. After that, aliquots of the lysis solution were added to RT Mastermix and subjected to the following conditions: first step 37°C (15 min), second step 50°C (5 min) and final step 98°C (5 min). Relative mRNA expression was evaluated by quantitative real‐time RT‐PCR using the KOD SYBR^🄬^ qPCR Mix (TOYOBO) with a thermal cycler (Bio‐Rad). The primers used for mRNA quantification are indicated in *Table* *S1*. PCR amplification and normalization of gene expression were performed as described previously.^15^, ^17^

### Laminin staining

A 4 μm‐thick section of the frozen tibialis anterior muscle was obtained; mounting compound was washed with phosphate‐buffered saline (PBS) and then blocked with 4% blocking agent (block ace, KAC Co., Ltd., Hyogo, Japan) in PBS at room temperature (RT) for 30 min. After that, samples were reacted with a primary antibody (anti‐laminin) at RT for 60 min. After that, samples were reacted with secondary antibody (anti‐rabbit‐IgG‐Alexa546) at RT for 60 min in a dark condition. Samples were observed by a microscope (BZ‐X700 microscope, Keyence, Osaka, Japan). The cross‐sectional area was quantified using the BZ‐X analyser (Keyence).

### Statistical analysis

All statistical comparison was conducted using R software (R Core Team, 2018; R Foundation for Statistical Computing, Vienna, Austria). The statistical comparison between the groups was performed using analysis of variance (ANOVA) followed by Tukey's multiple comparison test. A probability value of *P* < 0.05 was considered statistically significant.

## Results

### Alteration of myokine expression in skeletal muscle in chronic kidney disease mice

**Figure 2:**
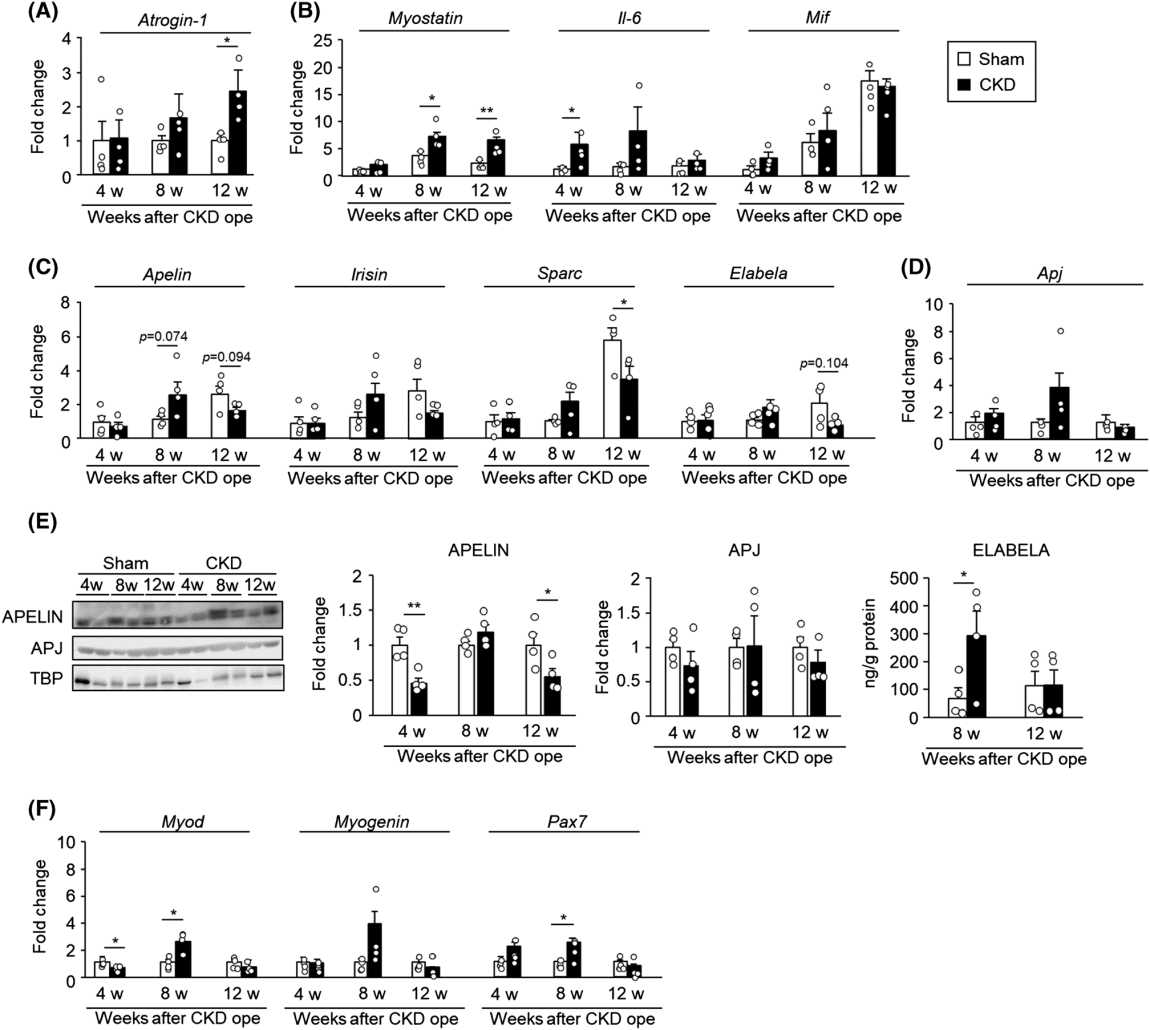
Progression of skeletal muscle atrophy and alteration of myokine expression in chronic kidney disease (CKD) mice. (A–D, F) mRNA expression in the gastrocnemius muscle of CKD mice was determined by real‐time RT‐PCR. mRNA expression of *Atrogin‐1*, *Apj*, *Myod*, *Myogenin* and *Pax7* was determined relative to the sham group that was used as a control. mRNA expression of *Myostatin*, *Il‐6*, *Mif*, *Apelin*, *Irisin*, *Sparc* and *Elabela* was determined relative to the sham‐operated group at 4 weeks. (E) Protein expression of APELIN and APJ or ELABELA was determined by western blot or enzyme‐linked immunosorbent assay (ELISA), respectively. Data are expressed as the means ± SEM (*n* = 4). *, **P < 0.05 or 0.01 compared with the respective sham‐operated group.

We investigated the change in muscle weight and myokine expression in skeletal muscles under CKD conditions (*Table* *1* and *Figure* *2*). Renal function was significantly decreased at 4 weeks after the 5/6‐Nx (*P* < 0.01) and was impaired until 12 weeks (*P* < 0.01) (*Table* *1*). Skeletal muscle weight showed a decrease at 4 weeks after the 5/6‐Nx and significantly decreased at 8–12 weeks (*Table* *1*). Consistent with the reductions in skeletal muscle weight, the expression of atrogin‐1 (a ubiquitin E3 ligase associated with skeletal muscle atrophy), myostatin and interleukin‐6 (IL‐6) was significantly increased in these stages (*Figure* *2* *A,B*). The mRNA and protein expression of apelin, elabela and their receptor, Apj, was evaluated. These expressions in GA muscle tended to decrease at 12 weeks after 5/6‐Nx; however, the serum concentration of apelin and elabela increased (*Table* *1* and *Figure* *2* *C–E*). The expression of irisin and SPARC (a factor associated with maintenance of skeletal muscle mass) was increased at 8 weeks after the 5/6‐Nx but showed a decrease at 12 weeks (*Figure* *2* *C*). The expression of mif showed an increase with aging but was not statistically significant in both groups (*Figure* *2* *B*). The expression of Apj, a receptor of apelin, and differentiation‐related genes such as myod, myogenin and pax7 was also transiently increased at 8 weeks after the 5/6‐Nx but showed a decrease at 12 weeks (*Figure* *2* *D,E*).

**Table 1:** Characteristics of mice after 5/6‐nephrectomy operation

		Body weight (g)	SCr (mg/dL)	BUN (mg/dL)	Indoxyl sulfate (μM)	GA (mg)	TA (mg)	SO (mg)	Apelin (pg/mL)	Elabela (pg/mL)
4 weeks	Sham	26.4 ± 2.0	0.25 ± 0.05	29.3 ± 2.6	7.8 ± 2.0	171.0 ± 12.7	54.8 ± 2.9	8.5 ± 0.9	‐	‐
	CKD	24.7 ± 0.7	0.52 ± 0.04**	41.8 ± 2.0**	33.7 ± 7.7**	151.0 ± 4.6	51.0 ± 1.4	7.3 ± 0.3	‐	‐
8 weeks	Sham	28.8 ± 0.9	0.13 ± 0.03	24.3 ± 1.3	7.1 ± 1.2	195.7 ± 1.4	66.5 ± 2.6	11.1 ± 0.8	860 ± 52	1895 ± 330
	CKD	27.1 ± 0.5	0.56 ± 0.09**	51.0 ± 2.2**	29.9 ± 4.5**	174.4 ± 5.7**	55.8 ± 1.8**	10.3 ± 0.5	1585 ± 133**	2522 ± 1068
12 weeks	Sham	30.3 ± 0.4	0.35 ± 0.06	37.4 ± 3.4	9.9 ± 1.5	176.8 ± 10.3	67.2 ± 5.3	9.7 ± 0.4	1742 ± 818	1876 ± 225
	CKD	28.0 ± 0.5	0.56 ± 0.06	52.9 ± 3.4**	24.5 ± 3.6*	163.2 ± 3.2	52.8 ± 1.4*	8.1 ± 0.3*	2823 ± 126	3530 ± 194**

*Note*: Data are expressed as means ± SEM (*n* = 4). Abbreviations: BUN, blood urea nitrogen; CKD, chronic kidney disease; GA, gastrocnemius; SCr, serum creatinine; SO, soleus; TA, tibialis anterior.

* *P* < 0.05.

** *P* < 0.01 compared with sham group.

### Relationship between the uraemic toxin and myokine expression in skeletal muscles of chronic kidney disease mice

**Figure 3:**
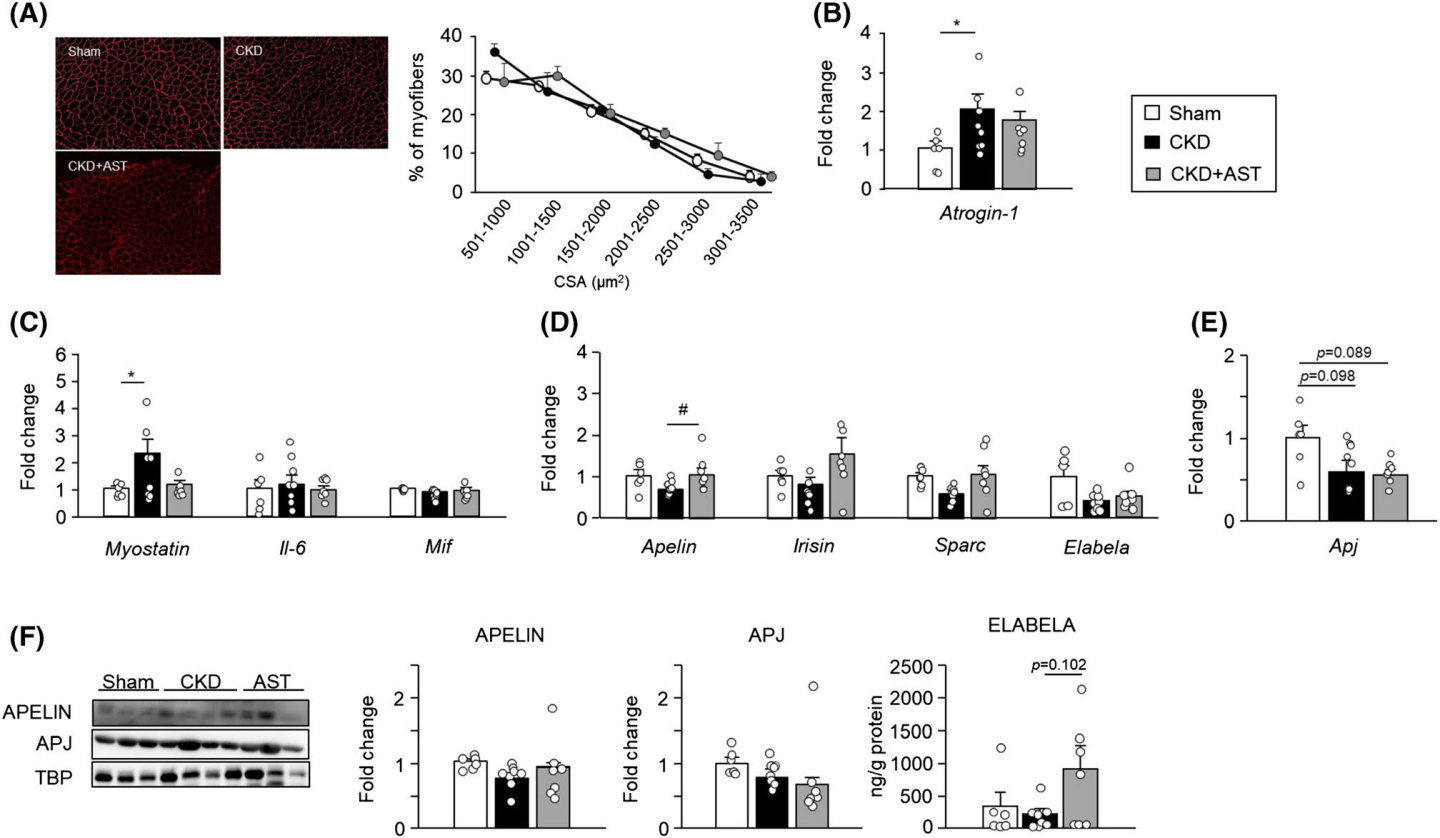
Relationship between uraemic toxins and myokine expression in skeletal muscles of chronic kidney disease (CKD) mice. (A) Cryosections of the tibial anterior muscle were immunostained with an anti‐laminin antibody to measure myofiber diameters. (B–E) mRNA expression of atrogin‐1, other myokines and Apj in the gastrocnemius muscle. (F) Protein expression of APELIN and APJ or ELABELA was determined by western blot or enzyme‐linked immunosorbent assay (ELISA), respectively. Data are expressed as the means ± SEM (*n* = 6–8). *P < 0.05 compared with each sham or indoxyl sulfate potassium (IS) (0 mM)‐treated group.^#^ *P* < 0.05 compared with CKD.

Next, we evaluated the relationship between the uraemic toxin and myokine expression in AST‐120‐administered CKD mice (*Table* *2* and *Figure* *3*). AST‐120 is an active charcoal adsorbent that reduces uraemic toxins in CKD by adsorbing uraemic toxins and their precursors in the intestinal tract and is used to evaluate the association of uraemic toxins.^15^ AST‐120 administration significantly inhibited uraemic toxin accumulation and skeletal muscle weight and muscle fibre size reduction but did not affect kidney function (*Table* *2* and *Figure* *3* *A*). The mRNA expression of atrogin‐1 in AST‐120‐administrated mice was not significant compared with sham mice (*Figure* *3* *B*). The increase of mRNA expression of myostatin in CKD mice was inhibited in AST‐120‐administrated mice (*Figure* *3* *C*). The reduction in the mRNA expression of apelin in GA muscle under CKD conditions was significantly inhibited by AST‐120 administration and protein expression levels were also slightly, but not significantly, increased (*Figure* *3* *D,F*). The mRNA expression of elabela was not changed by the administration of AST‐120; however, protein expression tended to increase (*Figure* *3* *D,F*). The decrease in Apj mRNA and protein expression was not restored by the administration of AST‐120 (*Figure* *3* *E,F*). The serum concentration of apelin and elabela was not changed by AST‐120 administration (*Table* *2*).

**Table 2:** Characteristics of AST‐120‐administrated chronic kidney disease mice

	Body weight (g)	SCr (mg/dL)	BUN (mg/dL)	Indoxyl sulfate (μM)	GA (mg)	TA (mg)	SO (mg)	Apelin (pg/mL)	Elabela (pg/mL)
Sham	28.9 ± 1.0	0.29 ± 0.05	35.4 ± 2.4	8.4 ± 1.4	171.3 ± 7.4	62.4 ± 4.7	9.2 ± 0.4	1610 ± 524	2891 ± 801
CKD	26.4 ± 0.7	0.42 ± 0.07	50.1 ± 2.7**	24.4 ± 2.5**	162.4 ± 2.4	52.7 ± 1.0*	8.0 ± 0.2	2316 ± 231	3597 ± 593
CKD + AST	28.7 ± 0.6	0.66 ± 0.08** ^,^ ^#^	57.2 ± 3.9**	14.5 ± 1.4^##^	176.6 ± 4.2	59.9 ± 0.7	9.9 ± 0.5^#^	2110 ± 255	4270 ± 651

*Note*: Data are expressed as means ± SEM (*n* = 6–8). Abbreviations: AST, AST‐120 (oral charcoal adsorbent); BUN, blood urea nitrogen; CKD, chronic kidney disease; GA, gastrocnemius; SCr, serum creatinine; SO, soleus; TA, tibialis anterior.

* *P* < 0.05.

** *P* < 0.01 compared with sham group.

# *P* < 0.05.

## *P* < 0.01 compared with CKD group

**Figure 4:**
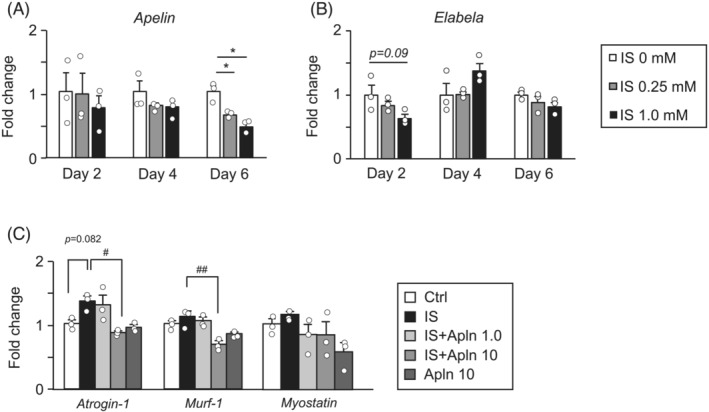
The effect of indoxyl sulfate potassium (IS) on apelin and elabela expression and efficacy of apelin in C2C12 cells. (A and B) The effect of IS (0, 0.25 and 1.0 mM) on apelin and elabela expression in C2C12 cells was determined by real‐time RT‐PCR. (C) The effect of apelin (1.0 or 10 nM) on IS (0.25 mM)‐induced skeletal muscle atrophy‐related genes expression in C2C12 cells was determined by real‐time RT‐PCR. Data are expressed as the means ± SEM (*n* = 3). *P < 0.05 compared with IS 0 mM.^#, ##^ *P* < 0.05 or 0.01.

To further investigate the relationship between uraemic toxin and apelin reduction, we evaluated its effect in cell culture experiments in vitro. We found that IS is related to decline of apelin and elabela mRNA expression in C2C12 myotube dose‐dependent manner (*Figure* *4* *A,B*). We also evaluated the mechanism of action of apelin in skeletal muscle atrophy induced by IS using C2C12 cells. Co‐treatment of apelin with IS significantly decreased atrogin‐1 expression compared with treatment with IS alone (*Figure* *4* *C*). In addition, treatment with apelin also decreased the expression of muscle RING‐finger protein 1 (MuRF‐1), a skeletal muscle atrophy‐related ubiquitin E3 ligase, compared with treatment with IS alone (*Figure* *4* *C*). Additionally, treatment with apelin also decreased myostatin mRNA expression (*Figure* *4* *C*).

### Effect of apelin on skeletal muscle atrophy in chronic kidney disease

**Figure 5:**
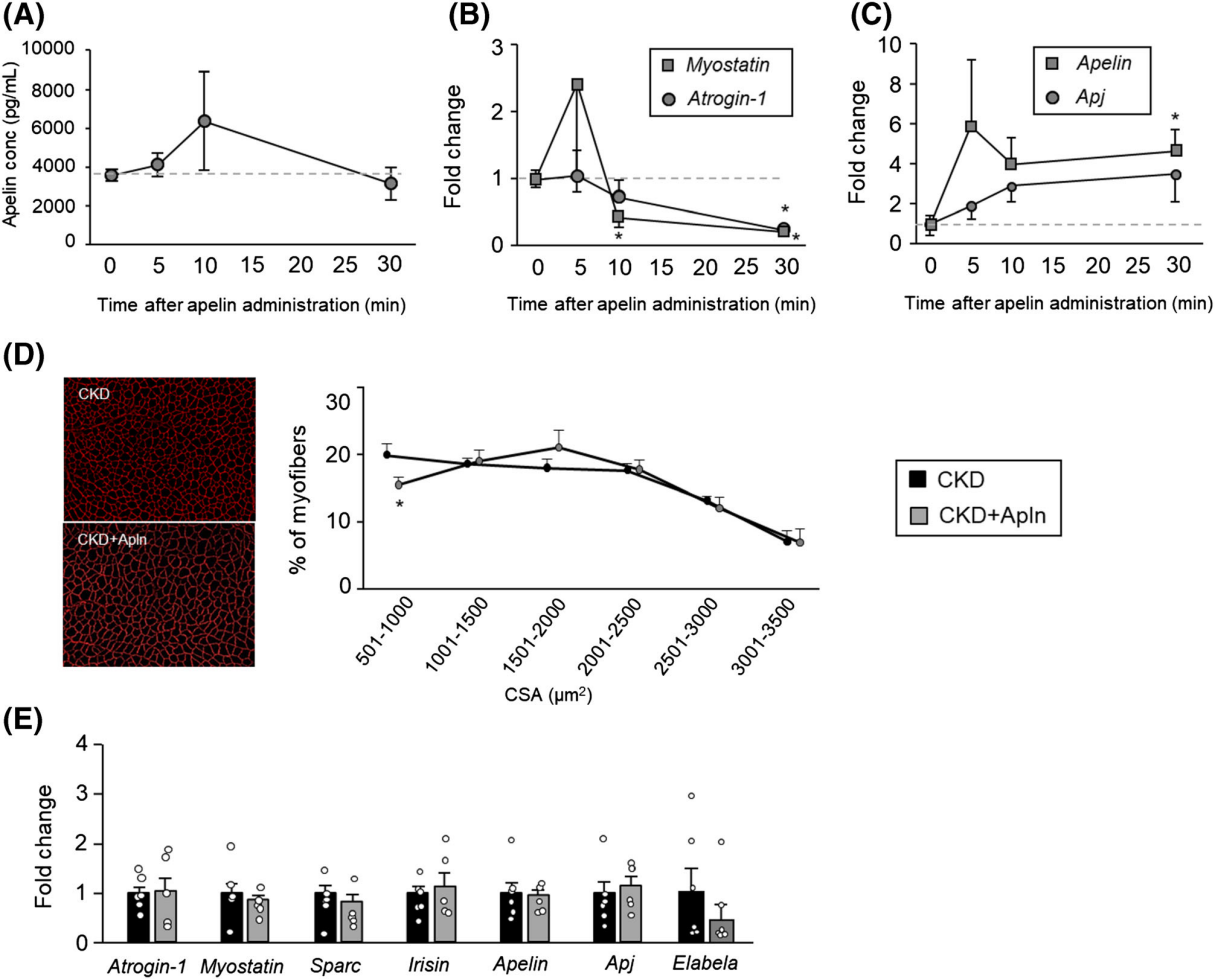
Effect of apelin on chronic kidney disease (CKD)‐induced skeletal muscle atrophy. (A) The serum concentrations of apelin after apelin administration (1 μmol/kg, intraperitoneally) in CKD mice were determined by enzyme‐linked immunosorbent assay (ELISA). (B and C) The change in mRNA expression in the gastrocnemius muscle after apelin administration (1 μmol/kg, intraperitoneally) in CKD mice. (D) Cryosections of the tibial anterior muscle were immunostained with an anti‐laminin antibody to measure myofiber diameters. (E) mRNA expression of *Atrogin‐1*, other myokines and *Apj* in the gastrocnemius muscle at 4 weeks after apelin administration. Data are expressed as the means ± SEM (*n* = 5–6). *P < 0.05 compared with the 0 min group after apelin administration or the CKD group.^#, ##^ *P* < 0.05 or 0.01 compared with indoxyl sulfate potassium (IS) alone, respectively.

To reveal the therapeutic potential of apelin for skeletal muscle atrophy under the CKD condition, we administered apelin (1.0 μmol/kg) 8 weeks after the 5/6‐Nx, which was considered to be a turning point for apelin expression alteration in the skeletal muscle (*Figure* *5*). We first evaluated the pharmacological effects of apelin after administration. The serum concentration of apelin was increased 10 min after apelin administration but rapidly decreased within 30 min (*Figure* *5* *A*). Apelin administration significantly decreased atrogin‐1 and myostatin mRNA expression (*Figure* *5* *B*), but not significantly inhibited at 4 weeks after apelin administration (*Figure* *5* *E*). In contrast, apelin mRNA expression was significantly increased following apelin administration, and Apj mRNA expression was also increased, but not significantly (*Figure* *5* *C*). Apelin administration significantly inhibited mass and cross‐sectional area reduction of skeletal muscle in CKD mice (*Table* *3* and *Figure* *5* *D*).

**Table 3:** Characteristics of apelin‐administrated chronic kidney disease mice

	Body weight (g)	SCr (mg/dL)	BUN (mg/dL)	Indoxyl sulfate (μM)	GA (mg)	TA (mg)	SO (mg)
CKD	28.4 ± 0.3	0.54 ± 0.06	52.9 ± 2.5	26.7 ± 1.8	176.1 ± 4.1	57.7 ± 1.1	10.6 ± 0.4
CKD + apelin	27.0 ± 0.8	0.57 ± 0.04	58.5 ± 3.4	23.0 ± 4.0	185.7 ± 3.3*	61.3 ± 1.1*	12.4 ± 0.2**

*Note*: Data are expressed as means ± SEM (*n* = 5–6). Abbreviations: BUN, blood urea nitrogen; CKD, chronic kidney disease; GA, gastrocnemius; SCr, serum creatinine; SO, soleus; TA, tibialis anterior.

* *P* < 0.05.

** *P* < 0.01 compared with sham group.

## Discussion

We investigated the association and therapeutic efficacy of administration of apelin and elabela, an endogenous Apj ligand and novel GPCR agonist, in skeletal muscle atrophy by CKD. Our results revealed that disturbance of the apelin–Apj system may be a factor in the progression of skeletal muscle atrophy in CKD. Administration of apelin ameliorates skeletal muscle atrophy in CKD mice; therefore, targeting the apelin–Apj system could be a promising therapeutic strategy for the management of skeletal muscle atrophy in CKD.

The apelin–Apj system has been reported to have therapeutic potential for various vascular diseases, such as CVD and diabetic angiopathy,^16^, ^18^ which have been observed in CKD patients. In addition to this efficacy, we uncovered new properties of apelin for CKD‐induced muscle atrophy. It has been reported that the reduction in mitochondrial biosynthesis and function, disturbance of the Akt pathway, and increased expression of muscle atrophy factors associated with uraemic toxins have been reported as a mechanism of CKD‐induced skeletal muscle atrophy.^15^, ^16^, ^17^, ^19^ The reduced muscle function observed in CKD may contribute to reduced endurance exercise and muscle weakness in early‐stage CKD patients. Apelin binds to the Apj and activates Akt and AMPK, thereby restoring mitochondrial biosynthesis and inducing hypertrophy.^12^ Our study confirmed that apelin gene expression and protein expression in skeletal muscle are reduced in CKD, but serum apelin concentration was not reduced, but rather increased. This suggests that the physiological expression of apelin in skeletal muscle may be due to an autocrine mechanism.

Reportedly, apelin expression in skeletal muscle is significantly decreased with age, but this decrease is not observed in the heart, liver or adipose tissue in mice.^12^ Our results indicated that apelin and Apj expression in skeletal muscle was transiently increased in the early stage of CKD, followed by a decrease in the late stage (*Figure* *2* *C*). This transient increase may be due to compensatory pathophysiological mechanisms related to CKD or arise due to differences in the temporal expression of factors associated with CKD progression. For example, induction of cardiotoxin‐administered skeletal muscle injury leads to increased apelin and Apj expression, which has been implicated in regeneration and repair processes following skeletal muscle injury.^12^ Consistent with a previous report,^12^ we confirmed a transient increase in the expression of Myod, myogenin and Pax7 genes that are related to skeletal muscle regeneration in CKD mice (*Figure* *2* *E*). We examined the effect of 4‐hydroxynonenal (4‐HNE) and the TNF‐α, uraemic toxins associated with CKD progression,^19^, ^20^ on the mRNA expression of apelin and Apj in C2C12 cells. Both uraemic toxins are reported to result in an increase in skeletal muscle catabolism and a decrease in mitochondrial function and biogenesis in CKD patients.^19^, ^21^ As a result, both factors increased apelin and Apj expression 24 h after treatment (*Figures* *S2* and *S3*). 4‐HNE reduced apelin and Apj expression at 72 h (*Figure* *S2*); however, TNF‐α increased the mRNA expression levels of the factors (*Figure* *S3*). It has been reported that the mRNA expression of apelin is increased in both mouse and human adipocytes by TNF‐α.^18^ Therefore, the roles of uraemic toxins associated with CKD progression on apelin expression may be different. The administration of AST‐120 slightly inhibited the reduction in apelin and elabela expression in GA muscle, but the serum concentration of each peptide and expression levels of Apj were unchanged (*Figure* *3* *D–F*). However, the decrease in skeletal muscle mass was markedly reversed by the administration of AST‐120. Uraemic toxins are known to inhibit the insulin/IGF‐1–PI3K–Akt pathway in skeletal muscles.^17^, ^22^ Administration of AST‐120 reduced uraemic toxins in serum (*Table* *2*) and muscle,^15^ which relieved the inhibition of the PI3K–Akt pathway, and slightly restored the expression levels of apelin and elabela, resulting in the suppression of muscle atrophy. In addition, IS treatment decreased the mRNA expression of apelin and elabela dose‐dependent manner in C2C12 myotubes, indicating that reduced apelin and elabela expression in CKD was partly caused by protein‐binding uraemic toxins. These results suggest that the decrease of apelin and elabela in skeletal muscles and the increase of uraemic toxins may be involved in muscle atrophy caused by CKD.

The control mechanism of apelin and elabela or Apj expression in CKD conditions may be explained by different mechanisms. AST‐120 inhibited the decrease in apelin expression in CKD but did not inhibit the decrease in Apj expression. Vinel et al. reported that apelin and Apj mRNA expression decreases with age in skeletal muscles, but the exact mechanism driving this is unclear.^12^ Other than skeletal muscle tissue, it has been reported that the expression of Apj in the renal artery is decreased in db/db mice.^23^ It has also been reported that the expression of Apj decreased in the myocardium of patients with chronic heart failure.^24^ However, the mechanism by which the expression of Apj decreases has not been clarified in these reports. Inflammation and oxidative stress are increased in db/db mice, chronic heart failure patients and 5/6‐nephrectomized mice, suggesting that these factors may be involved. However, cell culture experiments with TNF‐α and 4‐HNE showed different responses for Apj expression (*Figures* *S2* and *S3*). Thus, we hypothesize that these factors are intricately involved in the pathogenesis of CKD. In the present study, we considered that uraemic toxins adsorbed by AST‐120 have relatively little impact on Apj expression. The serum concentration of apelin and elabela was increased in CKD. Previous reports suggested that the plasma concentration of apelin was increased in obese patients, which were involved with insulin stimulation^25^ and TNF‐α.^18^ They reported that the mRNA amount in white adipocytes, heart and kidney was higher than in muscle (type of muscle was not determined) and brown tissue, and not detected in liver.^25^ Therefore, serum concentration of apelin may be unaffected by skeletal muscle expression level. In contrast, plasma elabela levels decreased in CVDs such as atrial fibrillation with hypertension^26^ and coronary heart disease.^27^ It was reported that TNF‐α was involved with the development of hypertension and coronary heart disease.^28^, ^29^ Elabela was detected in stem cells, kidneys, prostate, vascular endothelium and plasma.^30^ Differences in the plasma levels of apelin and elabela were reported in patients with gestational diabetes mellitus.^31^ Thus, control mechanisms of apelin, elabela and Apj in plasma and skeletal muscle tissue are involved in complex factors.

The blood level of apelin was increased after intraperitoneal administration and rapidly decreased within 30 min. Further, apelin administration significantly suppressed myostatin and atrogin‐1 expression but increased the apelin and Apj expression. However, after 4 weeks of apelin treatment, the expression of these genes did not change in the skeletal muscle. This may be a result of the disappearance of the pharmacological effect as the skeletal muscle sample was collected 24 h after the final administration. However, evaluation of the pharmacological effect soon after apelin administration indicated that the inhibitory effect on the skeletal muscle atrophy‐related gene expression was observed in a blood concentration‐dependent manner (*Figure* *5* *B,C*). A significant increase in skeletal muscle mass and myofiber area was observed in apelin administration (*Table* *3*); therefore, we confirmed that apelin had an inhibitory effect on skeletal muscle atrophy, resulting from CKD. In our experiment, we administered double the apelin dose described in a previous report.^12^ In our preliminary study, a half‐dose of apelin (0.5 μmol/kg/day), the same dose as described by Vinel et al., did not show a significant inhibitory effect on muscle atrophy in CKD mice (data not shown). This may be due to the faster progression of CKD‐induced skeletal muscle atrophy compared with the progression of age‐related sarcopenia. Additionally, systemic circulating apelin‐13 is quickly eliminated after intraperitoneal injection in mice, and systemic circulating apelin is also quickly eliminated in humans according to a previous report.^32^ It is necessary to improve the pharmacokinetics of apelin to enable its use as a drug for CKD management. For example, it was reported that the human IgG‐Fc region fused with recombinant apelin (Fc‐apelin‐13) improved blood retention times while retaining sufficient activity.^33^ Furthermore, the development of Apj agonists such as BMS‐986224 has progressed as a treatment for heart failure.^34^ In the present study, we used apelin‐13 as a ligand of Apj. However, other longer isoforms, such as apelin‐36 and apelin‐17, have higher affinities to Apj.^35^ In addition, the small‐molecule Apj ligands, such as AMG‐986 and AMG‐8123, also have affinities for Apj comparable with or higher than that of apelin, as well as a longer half‐life.^36^ Thus, apelin isoforms or the small‐molecule Apj ligands may be more effective than apelin against CKD‐induced skeletal muscle atrophy. However, the efficacy of apelin‐13 against CKD‐induced skeletal muscle atrophy demonstrated by our experiment presents a potential therapeutic strategy targeting the apelin–Apj system. Thus, in the development of new drugs against CKD‐induced sarcopenia, targeting the apelin–Apj system provides a promising therapeutic strategy.

This study had some limitations. First, we did not completely prove that the efficacy of apelin depended on the Apj or type I angiotensin II receptor (AT1R). Apj shows homology with AT1R (approximately 50%) and activation of Apj antagonizes AT1R signalling.^37^, ^38^ In general, the renin‐angiotensin‐aldosterone system (RAAS) is activated in CKD and is an important therapeutic target. In addition, angiotensin II induces skeletal muscle atrophy in mice^39^; therefore, it is not completely clear whether the present results depend on the direct therapeutic effect of apelin via Apj or the result of antagonizing RAAS signal via the Apj–AT1R axis. However, we believe that apelin is a promising therapeutic target for CKD‐induced sarcopenia.

**Figure 6:**
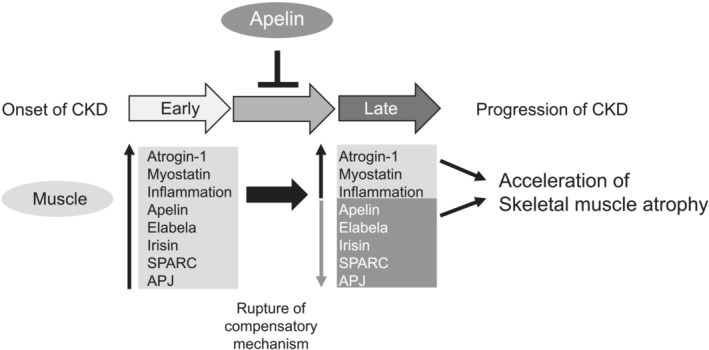
Proposed role of apelin in skeletal muscle atrophy in chronic kidney disease (CKD) and its therapeutic potential. In the early stage (approximately 8 weeks after 5/6‐nephrectomy) of CKD, myokine expressions such as myostatin, inflammation‐related, apelin, elabela, irisin and sparc were increased, and apj expression was also increased. In the late stage (>8 weeks after 5/6‐nephrectomy) of CKD, expression of apelin, elabela, irisin, sparc and apj decreased because of disruption of the compensatory mechanism, which may be the cause of the acceleration of skeletal muscle atrophy in CKD. Apelin administration transiently inhibits the increase of atrogin‐1 and myostatin expression and increases expression of apelin and Apj, which contributes to the amelioration of CKD‐induced skeletal muscle atrophy.

To summarize, we have shown that under CKD conditions, early compensatory changes in the apelin–Apj system and its subsequent failure of skeletal muscle tissue accelerate skeletal muscle atrophy in which uraemic toxins play an important role (*Figure* *6*). We also showed that suppression or reversal of these failures by exogenous Apj ligand supplementation inhibited CKD‐induced skeletal muscle atrophy. This is the first study to investigate the efficacy and usefulness of apelin against CKD‐induced skeletal muscle atrophy targeting the apelin–Apj system.

## Conflict of interest

The authors report no conflicts of interest.

## Supporting information


**Data S1.** Supporting Information

**Table S1** Primers list

**Figure S1.** **Purification and determination of synthesized apelin**. (A) HPLC chromatogram for purified apelin. (B) Mass spectrometry chromatogram for apelin.
**Figure S2. Effect of 4‐hydroxynonenal on apelin and Apj expression in C2C12 myotubes**. Effect of 4‐hydroxynonenal (100 μM) on mRNA expression of (A) apelin and (B) Apj was determined by real‐time RT‐PCR. Data are expressed as the means ± SEM (*n* = 3). *P < 0.05, **P < 0.01 compared with control.
**Figure S3. Effect of tumor necrosis factor‐α on apelin and Apj expression in C2C12 myotubes**. Effect of tumor necrosis factor‐α on mRNA expression of (A) apelin and (B) Apj was determined by real‐time RT‐PCR. Data are expressed as the means ± SEM (*n* = 3). **P < 0.01 compared with control.
