# Supplementary material for: The G protein‐coupled receptor ligand apelin‐13 ameliorates skeletal muscle atrophy induced by chronic kidney disease
Source: J Cachexia Sarcopenia Muscle. 2022 Dec 23;14(1):553–64. doi: 10.1002/jcsm.13159 (PMC9891924; doi:10.1002/jcsm.13159)
Supplement: Supplementary file 1 — Data S1. Supporting Information [file JCSM-14-553-s002.docx]

**The G protein-coupled receptor ligand apelin-13 ameliorates skeletal muscle atrophy induced by chronic kidney disease**

Yuki Enoki ^a,†,*^, Tomoya Nagai ^a,†^, Yuna Hamamura ^a^, Sumika Osa ^a^, Hideaki Nakamura ^b^, Kazuaki Taguchi ^a^, Hiroshi Watanabe ^c^, Toru Maruyama ^c^, Kazuaki Matsumoto ^a^

**Supplementary methods**

*Apelin synthesis*

Apelin (QRPRLSHKGPMPF) was synthesized on a 2-chlorotrityl chloride resin using standard Fmoc solid-phase methodology, which consisted of a manual reaction in a vessel and an automated reaction using a microwave synthesizer (Biotage® Initiator + Alstra, Tokyo, Japan). Fmoc-Phe-OH was linked to the resin (100−200 mesh, 1% divinyl benzene (DVB), 1.33 mmol/g) in DIPEA/DCM for 60 min at room temperature with gentle shaking. The resin was sequentially washed three times with DCM/MeOH/DIPEA (17:2:1), DCM, and MeOH, and dried in vacuo to obtain Fmoc-Phe-resin (0.40 mmol/g). H-Phe-resin, which was previously Fmoc-deprotected with 20% piperidine in DMF, was placed in a reaction vial. Subsequently, the protected amino acids were added to the resin in a stepwise manner. Each coupling reaction was performed using an Fmoc amino acid (5 eq., 0.5 M), coupling reagent DIC (5 eq., 0.5 M), and OXYMA (5 eq., 0.5 M) in DMF at 70°C for 5 min, instead of room temperature for 60 min for His coupling, followed by deprotection of Fmoc in 20% piperidine. After each coupling and deprotection step, the resin was washed with DMF. Apelin was cleaved from the resin using a mixture of TFA/TIPS/water/EDT (94:1.0:2.5:2.5 v/v/v/v). The resin was filtered, and the crude apelin was recovered by precipitation in diethyl ether. (A) The crude apelin was subsequently purified via reverse phase-high performance liquid chromatography (RP-HPLC) on a preparative C18 column (Cosmosil C18-MS-Ⅱ) using solvent A (0.1% TFA aq.) and a linear gradient from 15% to 30% solvent B (0.1% TFA in acetonitrile). (B) The apelin molecular weight determined by Matrix-Assisted Laser Desorption lonization Time of Flight Mass Spectrometry (MALDI-TOFMS, Autoflex III🄬, BRUKER, MA, USA) using 2,5-dihydroxybenzoic acid as a matrix.
