# Supplementary material for: The G protein‐coupled receptor ligand apelin‐13 ameliorates skeletal muscle atrophy induced by chronic kidney disease
Source: J Cachexia Sarcopenia Muscle. 2022 Dec 23;14(1):553–64. doi: 10.1002/jcsm.13159 (PMC9891924; doi:10.1002/jcsm.13159)
Supplement: Supplementary file 2 — Table S1 Primers list [file JCSM-14-553-s001.docx]

**The G protein-coupled receptor ligand apelin-13 ameliorates skeletal muscle atrophy induced by chronic kidney disease**

Yuki Enoki ^a,†,*^, Tomoya Nagai ^a,†^, Yuna Hamamura ^a^, Sumika Osa ^a^, Hideaki Nakamura ^b^, Kazuaki Taguchi ^a^, Hiroshi Watanabe ^c^, Toru Maruyama ^c^, Kazuaki Matsumoto ^a^

| Target gene | Forward | Reverse | bp |
| --- | --- | --- | --- |
| Tbp | 5’-GGGTATCTGCTGGCGGTTT-3’ | 5’-TGAAATAGTGATGCTGGGCACT-3’ | 75 |
| Atrogin-1 | 5’-CAGAGAGGCAGATTCGCAAG-3’ | 5’-GGTGACCCCATACTGCTCTC-3’ | 116 |
| Myostatin | 5’-CTGTAACCTTCCCAGGACCA-3’ | 5’-TCTTTTGGGTGCGATAATCC-3’ | 197 |
| Il-6 | 5’-TCTCTGCAAGAGACTTCCATCC-3’ | 5’-AGACAGGTCTGTTGGGAGTG-3’ | 126 |
| Mif | 5’- GCAAGCCCGCACAGTACAT -3’ | 5’- TGGCAGCGTTCATGTCGTAA-3’ | 222 |
| Apelin | 5’- GGAAGGGGTGTGGCGAAATA -3’ | 5’- TCTGACCTGGGGACTGAACA -3’ | 165 |
| Irisin | 5’-CACGCGAGGCTGAAAAGAT-3’ | 5’-CTTGTTGTTATTGGGCTCGTT-3’ | 191 |
| Sparc | 5’-ATGCAAATACATCGCCCCCT-3’ | 5’-TCTCAAAGTCTCGGGCCAAC-3’ | 221 |
| Apj | 5’-TCGGCTAAGGCTGCGAGTC-3’ | 5’-CGTCTGTGGAACGGAACAC-3’ | 101 |
| Myod | 5’-TGGCATGATGGATTACAGCG-3’ | 5’-GAGATGCGCTCCACTATGCT-3’ | 156 |
| Myogenin | 5’-TCCCAACCCAGGAGATCATT-3’ | 5’-TCAGTTGGGCATGGTTTCGT-3’ | 136 |
| Pax7 | 5’-CGTAAGCAGGCAGGAGCTAA-3’ | 5’-ACTGTGCTGCCTCCATCTTG-3’ | 152 |
| Murf-1 | 5’-TGTGCCAACGACATCTTCCA-3’ | 5’-AACGGAAACGACCTCCAGAC-3’ | 85 |
| Elabela | 5’-GCCATAGGATGTGGGGTGAG-3’ | 5’-CTCCTGCGAAAGCAGTTGTG-3’ | 275 |

**Table S1 Primers list**

Abbreviation: *Tbp* TATA box binding protein, *Il-6* interleukin-6, *Mif* macrophage migration inhibitory factor, *Sparc* secreted acidic cysteine rich glycoprotein, *Pax7* paired box 7, *Murf-1* muscle specific RING finger protein
