# Supplementary material for: The G protein‐coupled receptor ligand apelin‐13 ameliorates skeletal muscle atrophy induced by chronic kidney disease
Source: J Cachexia Sarcopenia Muscle. 2022 Dec 23;14(1):553–64. doi: 10.1002/jcsm.13159 (PMC9891924; doi:10.1002/jcsm.13159)
Supplement: Supplementary file 3 — Figure S1. Purification and determination of synthesized apelin. (A) HPLC chromatogram for purified apelin. (B) Mass spectrometry chromatogram for apelin. Figure S2. Effect of 4‐hydroxynonenal on apelin and Apj expression in C2C12 myotubes. Effect of 4‐hydroxynonenal (100 μM) on mRNA expression of (A) apelin and (B) Apj was determined by real‐time RT‐PCR. Data are expressed as the means ± SEM (n = 3). *P < 0.05, **P < 0.01 compared with control. Figure S3. Effect of tumor necrosis factor‐α on apelin and Apj expression in C2C12 myotubes. Effect of tumor necrosis factor‐α on mRNA expression of (A) apelin and (B) Apj was determined by real‐time RT‐PCR. Data are expressed as the means ± SEM (n = 3). **P < 0.01 compared with control. [file JCSM-14-553-s003.pptx]

## Slide 1
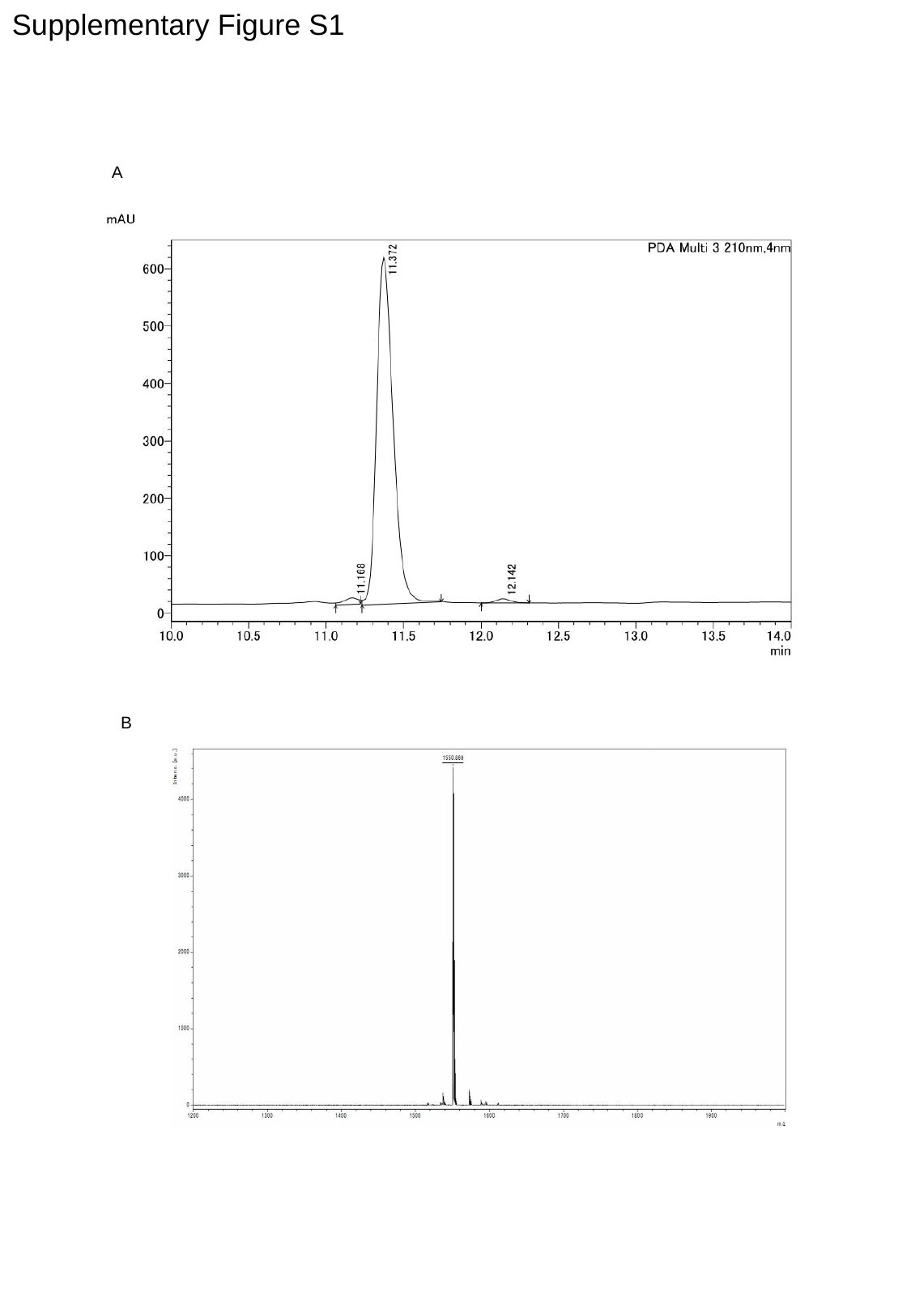

Supplementary Figure S1
A
B

## Slide 2
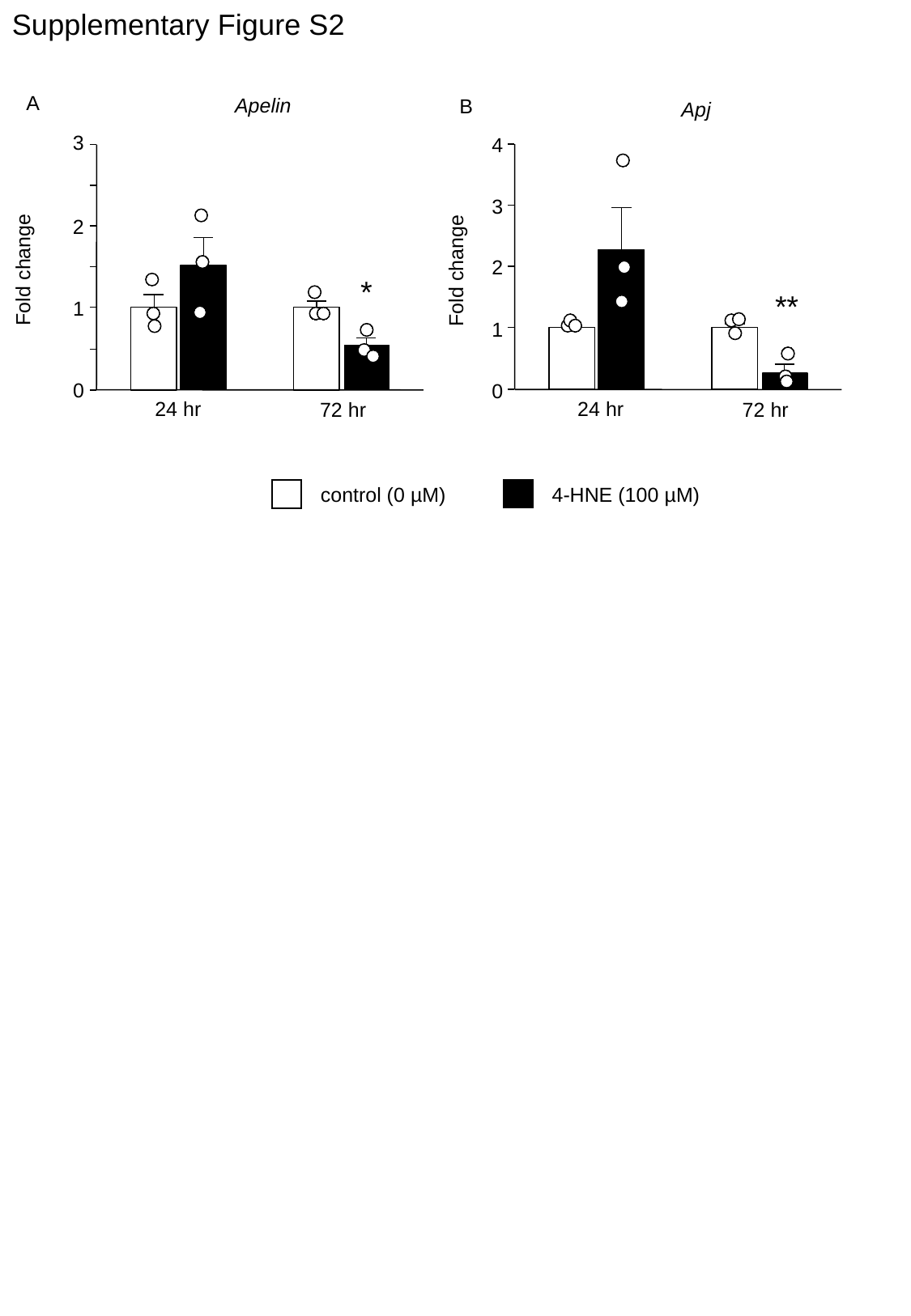

Supplementary Figure S2
A
Apelin
B
Apj
3
4
3
2
2
Fold change
Fold change
*
**
1
1
0
0
24 hr
24 hr
72 hr
72 hr
4-HNE (100 µM)
control (0 µM)

## Slide 3
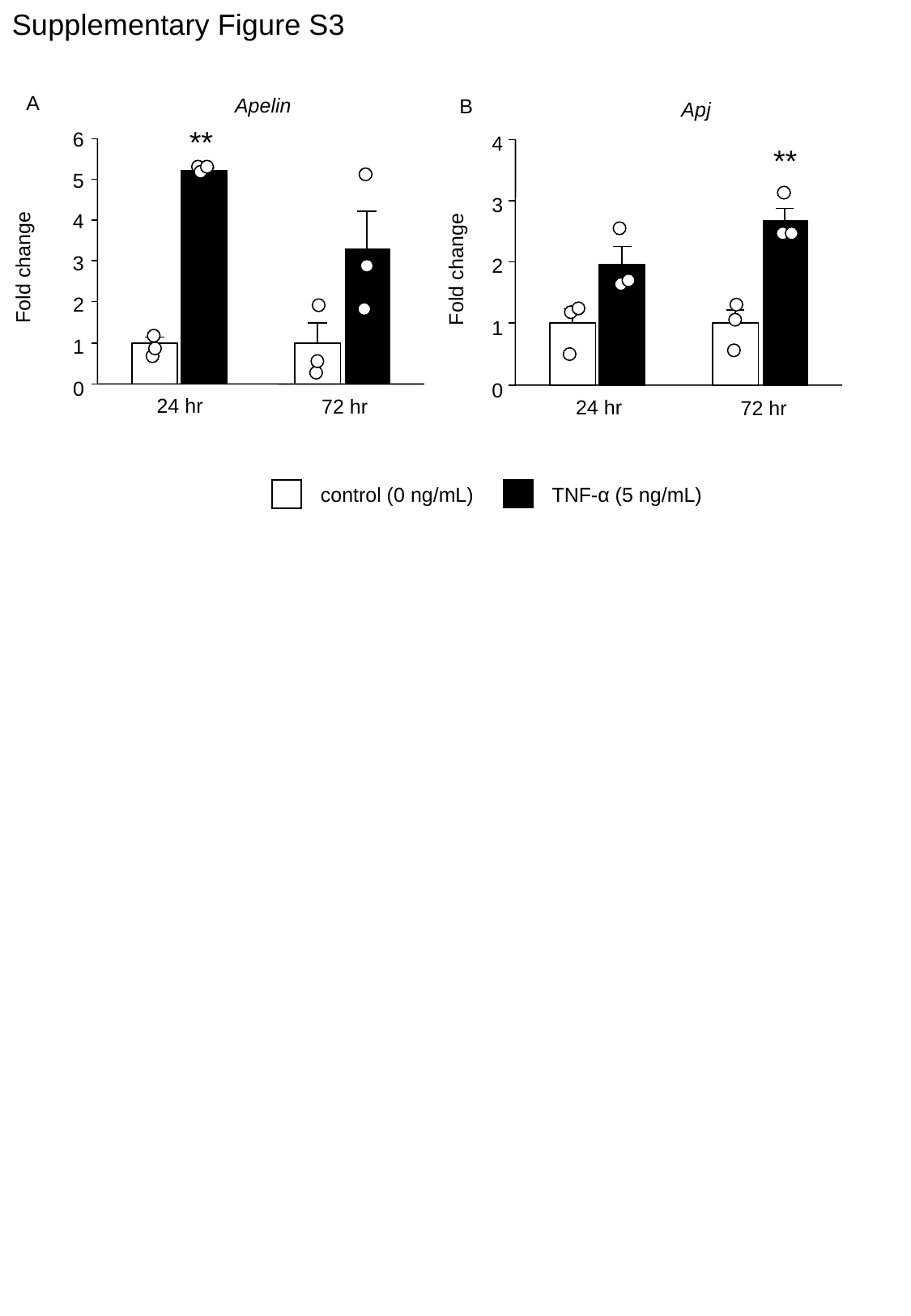

Supplementary Figure S3
A
Apelin
B
Apj
**
6
4
**
5
3
4
3
2
Fold change
Fold change
2
1
1
0
0
24 hr
72 hr
24 hr
72 hr
TNF-α (5 ng/mL)
control (0 ng/mL)
